# Effects of Unripe Black Raspberry Extract Supplementation on Male Climacteric Syndrome and Voiding Dysfunction: A Pilot, Randomized, Double-Blind, Placebo-Controlled Trial

**DOI:** 10.3390/nu15153313

**Published:** 2023-07-26

**Authors:** Su-Jin Jung, Eun-Ock Park, Soo-Wan Chae, Seung-Ok Lee, Ji-Wung Kwon, Jae-Hyung You, Young-Gon Kim

**Affiliations:** 1 Clinical Trial Center for Functional Foods, Biomedical Research Institute, Jeonbuk National University Hospital, Jeonju 54907, Republic of Korea; sjjeong@jbctc.org (S.-J.J.); eopark@jbctc.org (E.-O.P.); swchae@jbctc.org (S.-W.C.); solee@jbnu.ac.kr (S.-O.L.); 2 Clinical Trial Center for K-FOOD Microbiome, Biomedical Research Institute, Jeonbuk National University Hospital, Jeonju 54907, Republic of Korea; 3 Research Institute of Clinical Medicine, Medical School, Jeonbuk National University, Jeonju 54907, Republic of Korea; 4 Division of Gastroenterology and Hepatology, Department of Internal Medicine, Medical School, Jeonbuk National University, Jeonju 54896, Republic of Korea; 5 Berry & Bio Food Research Institute, Gochang, Jeonbuk 56417, Republic of Korea; kjwung@hanmail.net; 6 Department of Urology, Jeonbuk National University Hospital, Jeonju 54907, Republic of Korea

**Keywords:** aging male symptoms, IPSS, testosterone, BPH, metabolic syndrome, ADAM

## Abstract

Male climacteric syndrome (MCS) is a medical condition that can affect middle-aged men whose testosterone levels begin to decline considerably. These symptoms may include fatigue, decreased libido, mood swings, and disturbed sleep. MCS can be managed with lifestyle modifications and testosterone replacement. However, testosterone therapy may cause number of side effects, including an increased risk of cardiovascular issues. This study aims to evaluate the efficacy and safety of unripe black raspberry extract (BRE) against MCS and voiding dysfunction in men with andropause symptoms. A total of 30 subjects were enrolled and randomly assigned to the BRE group (n = 15) or the placebo group (n = 15). Participants were supplemented with 4800 mg BRE or placebo twice daily for 12 weeks. The impact of BRE was assessed using the Aging Male’s Symptoms (AMS scale), International Prostate Symptom Score (IPSS) and the IPSS quality of life index (IPSS-QoL). Additionally, male sex hormones, lipid profiles, and anthropometric indices were assessed 6 and 12 weeks after treatment. The AMS scores did not differ significantly between the two groups. In the BRE group, the total IPSS and IPSS-QoL scores decreased significantly after 12 weeks compared to baseline (*p* < 0.05), but there was no significant difference compared to the placebo group. However, a significant difference was observed in the IPSS voiding symptoms sub-score compared to the placebo group. Furthermore, LDL-C and TC levels were also significantly lower in the BRE group than in the placebo group (*p* < 0.05). Collectively, the study provides strong evidence supporting the safety of BRE as a functional food and its supplementation potentially enhances lipid metabolism and alleviates MCS and dysuria symptoms, limiting the development of BPH.

## 1. Introduction

Men are prone to chronic conditions such as aging males’ symptoms (AMS) and prostatic hyperplasia (PH), which can lead to urinary disorders such as lower urinary tract symptoms (LUTS), including intermittent urinary incontinence, dysuria, urgency, and nocturia and can negatively impact quality of life [1,2]. The decrease in function of Leydig cells and the expression of steroidogenesis-related enzymes with age are associated with a decline in testosterone levels, contributing to the development of male climacteric syndrome (MCS) [3]. Hence, testosterone replacement therapy is a common treatment for MCS, and it has been associated with potential side effects such as prostate cancer, benign prostatic hyperplasia, and cardiovascular disease [4]. Thus, it is important for middle-aged men to have safer alternative strategies that can naturally increase their testosterone levels, easing the symptoms of PH and dysuria. Testosterone replacement therapy with functional foods is often associated with efficacy and safety. Moreover, the majority of testosterone replacement therapy products have not been scientifically validated. Black raspberries are deciduous shrubs belonging to the Rosaceae family and native to North America. Black raspberries offer a range of potential health benefits due to their rich nutritional profile and unique phytochemical composition. It consists of ellagic acid, a polyphenol compound with antioxidant, antibacterial, anti-inflammatory, anti-aging, antidiabetic, and prostate and cholesterol suppression properties [5,6,7,8,9,10,11,12,13,14,15,16,17,18]. Furthermore, other phytochemicals such as anthocyanins, flavanols, and phenolic acids are reported to benefit health [9,10,11,12,13,14,16,17,18,19]. Nevertheless, the presence of these phytochemicals depends on the fruit’s maturity stage [9,10,11,12,13,14,16,17,18,19]. Previous reports suggest that unripe black raspberries comprise more phenolic compounds and vitamin C than ripe black raspberries, signifying their potential use as a functional component [9,19]. In Korea, unripe black raspberries have been traditionally used to treat prostate and urological diseases since ancient times. In support of their health benefits, several studies indicate that unripe black raspberries have beneficial pharmacological effects on prostate enlargement and andropause [8,20]. In addition, recent preclinical studies have demonstrated that the administration of unripe black raspberry extract (BRE) to animals promotes testosterone secretion via increased luteinizing hormone (LH) secretion, which demonstrates its potential to improve MCS parameters [8,20]. However, there are limited reports on its health benefits for MCS and voiding function in humans. To address this knowledge deficit, we conducted a randomized, double-blind, placebo-controlled exploratory trial to assess the safety and efficacy of BRE supplementation over 12-weeks in improving MCS and voiding dysfunction in men with andropause symptoms. The findings of this study may provide valuable insights into the potential benefits of unripe BRE in natural, safer, and continuous testosterone production to prevent PH and dysuria in men experiencing climacteric syndrome.

## 2. Materials and Methods

### 2.1. Test Supplement

**Figure 1:**
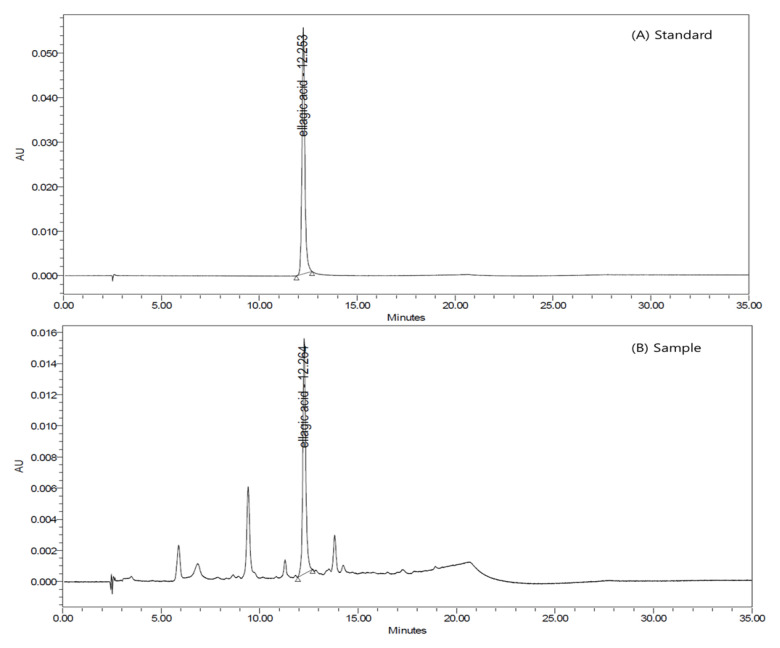
Representative high-performance liquid chromatography (HPLC) chromatograms of black raspberry extract (BRE).

To obtain the unripe black raspberry extract (BRE), 500 kg of dried unripe black raspberries, sourced from Gochang, Jeollabuk-do, Korea, were subjected to extraction using ethanol at a temperature of 80 ± 2 °C for a duration of 2 h. This mixture was then filtered using a 50 µm filter housing to obtain the initial extract. This extract, with a Brix concentration of 10–20%, was further concentrated at a temperature of 80 °C and subsequently lyophilized to yield the final BRE extract. Quantitative analysis of ellagic acid in BRE was performed using HPLC analysis. The average yield of the final BRE was 21.5 ± 0.9%, and the content of ellagic acid, an indicative component of BRE, was found to average 59.7 ± 1.8 mg/g. A standard stock solution was prepared by dissolving approximately 5 mg of ellagic acid (analytical standard, purity 95%, Sigma-Aldrich, MO, United States) in 25 mL of methanol. This solution was then diluted with methanol to create standard solutions with concentrations of 6.25, 12.5, 25, 50, 100, 150, and 200 μg/mL. After preparation, the stock and standard solutions were refrigerated at 4 °C until further use. About 0.1 g of BRE was placed into a 100 mL volumetric flask. To this, 10 mL of a pre-processing solvent (composed of 20 mL HCl, 20 mL tertiary distilled water, and 60 mL ethanol) was added, and the mixture was thoroughly dissolved. This mixture was then hydrolyzed for 3 h in a reflux extraction device at 90 °C, and subsequently cooled to room temperature. The hydrolyzed solution was transferred to a 50 mL volumetric flask, further dissolved with methanol, and then filtered through a 0.45 μm syringe filter before use. The ellagic acid content in BRE was determined using an ACQUITY UPLC H- Class HPLC instrument from Waters. The amount of ellagic acid was calculated from a calibration curve constructed from the peak areas at each concentration of the standard solution. Chromatograms were obtained using a Shiseido CAPCELL PAK C_18_ UG column (4.6 × 250 mm, 5 μm) as the stationary phase. The mobile phase consisted of two solvents: solvent A was water with 0.1% phosphoric acid added, and solvent B was methanol (J.T. Baker, AVANTOR, USA). The mobile phase was run at a flow rate of 1 mL/min. Detection was carried out by measuring the UV absorbance at a wavelength of 370 nm. Each sample injection volume was 20 μL. The linearity, detection limit (DL), and quantitation limit (QL) of ellagic acid were determined using UPLC. A calibration curve was prepared using stepwise diluted standard solutions, yielding a high coefficient of determination (R²) of 0.999983, indicating strong linearity in the concentration range of 6.25 to 200 μg/mL. The DL, where the signal-to-noise (S/N) ratio was 3.3, was found to be 0.1 μg/mL, while the QL, with an S/N ratio of 10, was determined to be 0.3 μg/mL. This implies that, for the sample, the DL and QL are 0.1 μg/mL and 0.3 μg/mL, respectively. The ellagic acid standard and BRE were examined using UPLC to study the separation of ellagic acid. Comparing the retention times from the chromatograms helped confirm the successful isolation of the ellagic acid peak. Both solutions displayed the ellagic acid peak at about the same time (around 12 min), confirming its identity (Figure 1). The analysis showed that the ellagic acid peak in the test solution was distinctly separated from other peaks, verifying its selective and accurate measurement.

All extraction procedures were performed by the Berry and Bio Food Research Institute in Gochang, Jeollabuk-do, Republic of Korea, and the test BRE product was provided in the form of yellow-brown powder tablets. The placebo product contained maltodextrin and was identical to the test product in appearance, flavor, and weight (Table 1). Next, in a preclinical investigation [8,20], benign prostatic hyperplasia (BPH) was induced in 10-week-old Sprague Dawley (SD) male rats through a daily subcutaneous injection of 3 mg/kg of testosterone propionate (Samil Pharmaceutical Company, Seoul, Korea) for 6 weeks. Following this, 300 mg/kg of BRE was administered orally for an additional 6 weeks [8]. This resulted in increased testosterone levels, reduced prostate weight, and changes in dihydrotestosterone (DHT) levels [8]. Similarly, older rats (24-week-old male rats) were orally administered with various concentrations of BRE (100, 200, 300, 400 mg). The 300 mg/kg BRE dosage showed the highest increase in total testosterone and LH levels. Furthermore, sperm motility increased at 300 mg/kg [20]. Based on these results, the daily recommended intake of BRE for humans was estimated to be 3000 mg per day.

### 2.2. Subjects

Volunteers aged 40 to 75 years were recruited from the Clinical Trial Center for Functional Foods (CTCF2) at Jeonbuk National University Hospital (JBUH) through internal advertising, including internet postings on departmental home pages, brochures, and posters, between 20 August 2019 and 2 December 2020. The study was approved by the Institutional Review Board (IRB) of JBUH (IRB No.2019-04-063) and registered with the Clinical Research Information Service of the Republic of Korea (approval number: KCT0008306). The clinical trial was conducted in compliance with the Helsinki Declaration and the Korean Good Clinical Practice (KGCP) provisions. The participants signed a consent form and underwent a screening assessment. A total of 30 participants were selected after undergoing screening tests, including questionnaires, physical examinations, and laboratory examinations. The selected participants were enrolled 3 weeks before receiving the test supplement. The criteria for selecting subjects were as follows: (1) men aged 40 to 75 years; (2) MCS, as determined by an aging males’ symptoms (AMS) scale score of 27 or higher; (3) based on the androgen deficiency in aging males (ADAM) questionnaire where participants’ answers for questions 1 or 7 are “yes,” or 3 or more of the other questions are “yes”; (4) total testosterone (TT) level between 2.51 ng/mL and 10.63 ng/mL; and (5) volunteers with understanding on the objectives of the study and agreeing to adhere by study rules throughout the study.

Exclusion criteria were (1) participants who had received testosterone or testosterone inhibitors within the previous 2 months; (2) participants who had received intramuscular testosterone within the previous 6 months; (3) participants with a body mass index (BMI) of 30 kg/m^2^ or more; (4) participants with a total prostate-specific antigen (T-PSA) level of 4.0 ng/mL or more; (5) participants with hyperprolactinemia, defined as blood prolactin levels 3 times or more of the upper limit of the reference range; (6) participants with severe acute or chronic cardio-cerebrovascular disease, metabolic disease, liver and biliary tract disease, pancreatic disease, muscle disease, neurological disease, mental disease, endocrine system disease, immune disease, kidney disease, malignant tumor, lung disease, urinary system diseases, and other diseases; (7) participants diagnosed with diabetes or with an HbA1c of 6.5% or more; (8) participants with hypotension below 90/50 mmHg or uncontrolled hypertension above 160/100 mmHg; (9) participants who had taken medication or received treatment to improve and treat male menopause, sexual function, impotence, enlarged prostate, etc., in the previous 2 months; (10) participants who had taken functional foods and herbal medicines to improve male menopause, sexual function, impotence, enlarged prostate, etc., in the previous 2 weeks; (11) participants with a history of hypersensitivity or clinically significant hypersensitivity to components of drugs and products for human application; (12) participants with a history of gastrointestinal diseases (e.g., Crohn’s disease, etc.,) or gastrointestinal surgery (except for simple appendectomy or hernia surgery) that may affect the absorption of products for clinical trial; (13) participants with a history of alcoholism or substance abuse; (14) participants who had been diagnosed with antipsychotics or had received drug treatment in the previous 2 months; (15) participants who had participated in other clinical trials in the previous 2 months; (16) participants with abnormal laboratory test results, including AST, ALT > 3 times or more of the upper limit of the reference range and serum creatinine > 2.0 mg/dL; and (17) participants deemed inappropriate for the clinical trial by the investigator.

### 2.3. Study Design

This study was a 12-week, randomized, double-blind, placebo-controlled trial conducted to assess the safety and efficacy of BRE against MCS and voiding dysfunction. A total of 30 subjects participated in the study, with 15 individuals assigned to the BRE and 15 to the placebo group. Subjects who met the selection criteria were assigned to the BRE group or placebo group based on an allocation code generated using a block-based random assignment method. Supplementary Figure S1 illustrates the schematic representation of the volunteer recruitment process. The study participants consumed the test products with water thrice daily, 30 minutes after breakfast, lunch, and dinner. The BRE group consumed a total of 4.8 g per day (3 doses, 6 tablets per day, each with 500 mg of BRE and 1800 mg of excipient). Similarly, the placebo group consumed a total of 4.8 g per day (3 doses, 6 tablets per day, each with 800 mg of maltodextrin). Table 1 lists the detailed composition of the test product (BRE) and placebo tablets. The participants received a baseline evaluation of their health status at the first visit (week 0), and then visited CTCF2 every 6 weeks (second visit, week 6; third visit, week 12) to examine vital signs, drug dosage, change in medical condition, and adverse reactions. The intake of test capsules was monitored by the investigator, who counted the remaining tablets to monitor compliance. All study participants, physicians, nurses, and clinical staff were blinded to study assignments. All the participants were informed not to use any supplements or traditional medicine, to avoid excessive exercise, and to have enough sleep 1 day before the assessment days. 

### 2.4. Randomization

The randomization module of SAS^®^ system version 9.3 (SAS Institute, Cary, NC, USA) was used to create a random assignment table using a sequence of random A and B numbers before the beginning of the trial. Registered subjects were randomly assigned in a 1:1 ratio to either the BRE or placebo groups before their first visit. According to the randomization list, test product tablets were assigned a randomization number, and the list was kept confidential throughout the study.

### 2.5. Outcome Measurement

A set of tests were conducted at predetermined time intervals (week 0, week 6, and week 12) to determine the safety and effectiveness of test products. On the day of evaluation, subjects arrived at CTCF2 on an empty stomach for urine and blood tests. All the urine and blood samples were stored at −80 °C until analysis.

### 2.6. Primary Outcome

The evaluation of AMS score via questionnaire was set as a primary outcome of the study. The AMS questionnaire was completed at the first visit (week 0), second visit (6th week), and third visit (12th week). The questionnaire included a total of 17 items and 3 sub-domains: mental symptoms (5 items), physical symptoms (7 items), and sexual symptoms (5 items). Each item was rated on a Likert 5-point scale, with higher scores indicating more severe symptoms. A score of 17–26 indicates no symptoms, a score of 27–36 is mild, a score of 37–49 is moderate, and a score of 50 or more indicates high severity [21,22,23]. 

### 2.7. Secondary Outcome

#### 2.7.1. International Prostate Symptom Score (IPSS)

The IPSS was measured as a secondary outcome in this study. Here, the IPSS questionnaire was completed by the subjects on their evaluation visits on week 0, week 6, and week 12. The IPSS consists of 7 items related to urinary symptoms, such as incomplete emptying and nocturia. The severity of the symptoms is categorized as mild, moderate, or severe based on the total score, which ranges from 1 to 35, with 0–7 indicating mild, 8–19 moderate, and 20–35 severe symptoms. Additionally, the IPSS quality of life sub-scale (IPSS-QoL) was measured, which measures the impact of urinary symptoms on the quality of life. The total IPSS was calculated by summing the scores of the 7 items, and the sub-scores of storage and urination symptoms were evaluated separately [24]. 

#### 2.7.2. Measurement of Sex Hormones

Total testosterone (TT), sex hormone binding globulin (SHBG), free testosterone (FT), and bioavailable testosterone (BT) were measured on all visits by the subjects. Specifically, blood was drawn from the brachial vein between 7:00 and 11:00 am to measure TT and SHBG. FT and BT were calculated using a calculator (http://www.issam.ch/freetesto.htm (accessed on 1 September 2019)) based on the measured values of TT, SHBG, and albumin. Follicle-stimulating hormone (FSH) and luteinizing hormone (LH) levels were measured on the third visit (week 12).

#### 2.7.3. Lipid Profiles and Anthropometric Index

Lipid profiles, including total cholesterol (TC), triglyceride (TG), and high-density lipoprotein-cholesterol (HDL-C), were measured at first (week 0) and third (week 12) visits. Low-density lipoprotein-cholesterol (LDL-C) was calculated using the Friedewald formula unless the TG concentration exceeded 400 mg/dL. If LDL-C was above 400 mg/dL, LDL-C was measured directly. Anthropometric measurements such as height, weight, body mass index (BMI), body fat mass (BFM), percentage body fat (PBF), fat-free mass (FFM), waist circumference (WC), hip circumference (HC), and waist–hip ratio (WHR) were measured using Inbody 720 (Biospace, Seoul, Korea) on first and third visits.

### 2.8. Safety Outcome Measurements

The safety of the study was evaluated by monitoring the clinical conditions and adverse events of the participants, which were recorded on the case report form. Vital signs, electrocardiograms, and laboratory tests were conducted to assess safety. In addition to relying on the subjects’ reports, physicians proactively inquired about potential health issues at each patient visit to ensure the safety of participants. This approach allowed for a comprehensive recording of any adverse reactions. Additionally, systolic and diastolic blood pressure, pulse rate, vital signs, and a physical examination were undertaken to ensure the safety of the subjects. Further, hematological examinations were conducted to measure white blood cell (WBC), red blood cell (RBC), hemoglobin, hematocrit, and platelet count. Blood biochemical tests were performed to measure bilirubin, alkaline phosphatase (ALP), gamma-glutamyl transferase (GGT), alanine transaminase (ALT), aspartate transaminase (AST), blood urea nitrogen (BUN), glucose, and creatinine levels. Liver and renal function tests were also conducted to evaluate safety. All the laboratory profiles of the subjects are shown in the Supplementary Table S1. 

### 2.9. Evaluation of Diet and Physical Activity

The subjects were instructed to maintain their usual lifestyle, dietary intake, and physical activity during the study period. However, they were asked to maintain a dietary record as per the guidelines set by the professional nutritionist. All the dietary intakes were assessed based on the records and information collected through face-to-face interviews on assessment days (weeks 0, 6, and 12). On the basis of these recorded and retrieved diet records, the average daily dietary intake was calculated and presented using the Can-Pro 4.0 software program (Korean Nutrition Society, Seoul, Republic of Korea). Physical activity was determined as per metabolic equivalent task (MET) assessment using the global physical activity questionnaire (GPAQ) [25]. MET value represents the relative proportion of working metabolic rate to metabolic rate at rest. 

### 2.10. Statistical Analysis

All the statistical analyses were performed with the help of SAS^®^ version 9.4 (SAS Institute, Cary, NC, USA) for all statistical analyses. Full analysis set (FAS) and per protocol (PP) analyses were performed in this study. Continuous variables were presented as mean ± standard deviation (SD), and categorical variables were analyzed using the Chi-square test (Fisher’s exact test) and the Wilcoxon signed-rank test for continuous variables. Furthermore, change in parameters that influenced the efficacy were analyzed using the Wilcoxon signed rank test or RM-ANOVA (repeated measures analysis of variance). Baseline and demographic variables were calibrated for heterogeneous evaluation with covariates for analysis of covariance (RM-ANCOVA) testing. Significance was set at a *p*-value of <0.05.

## 3. Results

### 3.1. Demographic Characteristics of Participants

The general characteristics of the individuals who participated in this study are shown in Table 2. The age, height, weight, birth count, BMI, vital signs, FT, BT, FSH, LH, HbA1c, alcohol consumption, smoking status, etc., of the participants were recorded as a baseline index. All the parameters, including total testosterone and prolactin levels, were similar in BRE and placebo groups (*p* > 0.05). One of the 15 registered subjects from the BRE group withdrew their consent. The selection process of subjects for the study is outlined in the Supplementary Figure S1. The compliance with the test products in the BRE and placebo groups was 97.1 ± 3.4% and 95.5 ± 5.3%, respectively. 

### 3.2. Diet Intake and Physical Activity

The nutrient intake of the subjects in BRE and placebo groups during the 12-week intervention period is presented in Table 3. There were no significant differences between the BRE and placebo group daily consumption of calories, carbohydrates, proteins, fats, and fibers (*p* > 0.05) before and after the completion of the intervention period. Furthermore, there was no significant difference in the corrected results, as the difference in the baseline carbohydrate intake between the groups was not significant (*p* > 0.05). Some of the parameters may be affected by the level of physical activity, but the analysis of metabolic equivalents (MET) reveals that neither the BRE nor placebo groups experienced any significant changes (*p* > 0.05) in physical activity.

### 3.3. Efficacy Evaluations

#### 3.3.1. Primary Outcome

In this study, AMS scores of the groups are calculated based on the subject’s responses to the AMS questionnaire and are listed in Table 4. The total AMS scores in the BRE group decreased considerably after supplement consumption for 12 weeks compared to baseline (*p* = 0.037). Similarly, total AMS scores in the placebo group decreased significantly (*p* = 0.001). Moreover, there was no significant difference observed between the two groups (*p* > 0.05). 

#### 3.3.2. Secondary Outcome

All the assessments on IPSS, ISS-QoL, voiding symptoms, TT, SHBG, FT, BT, FSH, and LH are listed in Table 4. In this study, total IPSS scores decreased in the BRE and placebo groups, but the decrease in the BRE group was significantly more significant. In addition, the BRE group showed a significant decrease in the urination symptoms sub-score after 6 and 12 weeks of consumption compared to baseline consumption (*p* = 0.005, *p* = 0.023). Further, sub-scores of voiding symptoms significantly differed from the placebo group (*p* = 0.021, *p* = 0.039). The IPSS-QoL score significantly reduced after 12 weeks of BRE consumption (*p* = 0.047), but there was no significant difference in the placebo group. Furthermore, no significant difference was observed in sex hormone indices in BRE and placebo groups. 

Anthropometric indices and lipid metabolism indicators such as TC, TG, HDL-C, LDL-C, and TC/HDL-C are listed in Table 5. There was no significant difference in all the anthropometric indices (*p* > 0.05). TC levels decreased significantly in the BRE group (*p* = 0.079), while TC increased in the placebo group (*p* = 0.044), presenting a statistically significant difference (*p* = 0.011) between the two groups. Similarly, LDL-C levels decreased significantly in the BRE group (*p* = 0.049), while LDL-C increased in the placebo group (*p* = 0.083), indicating a statistically significant difference (*p* = 0.007) between the two groups. Furthermore, HDL-C levels significantly differed between the two groups (*p* = 0.012). The ratio of LDL-C and HDL-C, an indicator of cardiovascular risk, was significantly reduced in the BRE group (*p* = 0.017), whereas the placebo group indicated a marginal reduction in LDL-C and HDL-C ratio. The ratios of TC and HDL-C indicated a minimal reduction.

### 3.4. Safety Parameters

Hematological, blood-biochemical, and urine analyses were performed at baseline and after 12 weeks of treatment. The results of blood counts, biochemical tests, and diagnostic tests (hematology, blood chemistry, and urine) in the BRE group showed no statistically significant difference before and after intake. Furthermore, systolic and diastolic blood pressure, pulse rate, body temperature, and other vital signs remained within the normal range. All the laboratory profiles of the subjects are shown in Supplementary Table S1.

### 3.5. Adverse Events

All study participants’ adverse reactions to the test product or placebo were documented at each visit. No serious adverse events were reported throughout the trial. However, five mild adverse events were reported by four subjects. The reported adverse events in the BRE group were upper respiratory infection (one case), dysuria (one case), elevated T-PSA (one case), heartburn (one case), and oral leukoplakia (one case). However, the relationship with the investigational product has been ruled out for all five mild adverse events. The incidence rate of adverse events did not differ significantly between the BRE and placebo groups (*p* > 0.05).

## 4. Discussion

This study is the first placebo-controlled, randomized, double-blind clinical trial to determine the effect of BRE on MCS and voiding dysfunction. Based on the preclinical investigation, the daily recommended intake of BRE for humans was estimated to be 4.8 g (as to BRE 3 g) per day. Safety-wise, no clinically significant adverse events or changes in the body were observed during this study. Therefore, BRE was considered safe for human consumption. In this trial, the consumption of 4.8 g BRE per day for 12 weeks in men with climacteric syndrome showed improved lipid profiles and voiding symptoms. 

BPH is a progressive disease prevalent in older men, and its occurrence increases with age [26]. It poses risks such as acute urine retention, decreased renal function, urinary tract infections, and urinary incontinence, mainly due to prostate gland enlargement [27]. In addition, LUTS is often associated with BPH and worsens urine storage and voiding function, making normal urination difficult and adversely affecting the quality of life. Moreover, BPH is strongly associated with testicular androgens and metabolic syndrome. Adopting a healthy lifestyle, including smoking cessation, moderate alcohol consumption, reduced intake of animal products, adequate sleep, and regular exercise, are crucial in preventing LUTS. Furthermore, MCS is a medical condition that can affect middle-aged and older men whose testosterone levels begin to decline considerably. These symptoms may include fatigue, decreased libido, mood swings, and disturbed sleep. MCS can be managed with lifestyle modifications and testosterone replacement. Interestingly, lower testosterone levels and a faster decline in testosterone levels are associated with prostate cancer and BPH development. The close relationship between MCS, BPH, and LUTS suggests that testosterone therapy may serve as a common treatment for all these health issues. However, testosterone replacement therapy has potential adverse effects, including cardiovascular disease [2]. 

The significance of functional diets in the management of a variety of medical conditions has grown substantially. These specialty foods contain bioactive compounds offering therapeutic benefits beyond basic nutrition. Berries are rich in flavonoid antioxidants, which may assist in protecting testosterone-producing cells from damage and enhancing testosterone production [28]. Specifically, blueberries are an excellent source of ellagic acid, an antioxidant known for improving testosterone levels [29,30,31]. Additionally, ellagic acid ameliorates the androgen-mediated prostate hyperplasia induced by AR signaling and STAT3 activation in animal and cell models of TP-induced BPH [31]. Hence, this clinical trial supplemented BRE rich in ellagic acid for 12 weeks to improve the BPH and urinary functions. The study outcomes were evaluated with the help of the AMS score, total IPSS scores, and IPSS quality of life index, and by measuring the sex hormone and metabolic parameters. Interestingly, AMS score did not significantly improve with BRE supplementation compared to a placebo group. Further, the primary observations of IPSS scores suggested that BRE supplementation improved the voiding function and lipid metabolic indices throughout the intervention period. 

The BRE group experienced a significant reduction in the IPSS voiding sub-score at 12 weeks, with noticeable improvement seen as early as 6 weeks, whereas there was no significant difference between groups in terms of the IPSS storage sub-score. Specifically, the BRE group’s total IPSS score improved from moderate (11.4 ± 3.3 points) to mild (7.9 ± 6.4). These results suggest that continuous BRE consumption for 12 weeks markedly improved voiding symptoms and quality of life. Contrarily, these subjects exhibited potential risk factors for BPH, with complaints of moderate dysuria symptoms, and their IPSS scores, ranging from 8 to 19, indicated voiding dysfunction. Similarly, IPSS scores indicated that supplementation with pumpkin seed extract improved dysuria symptoms [32,33,34]. A previous study reported that metabolic syndrome (Mets) in benign prostatic enlargement patients (average age 67 years) significantly increases the IPSS storage sub-score and the risk of storage symptoms in BPE patients [35]. These observations indicate the reliability of IPSS scores. Clinically, alpha-blockers are advised to relieve urinary symptoms in BPH patients [36,37]. However, if the improvement of urination symptoms is insignificant or if the prostate volume is 30 cc or more, PSA is 1.5 ng/mL or more, or if the digital rectal examination shows prostatic hyperplasia, the effect can be expected through concomitant administration of 5-alpha reductase inhibitors (5ARIs) [38]. Here, BRE supplementation improved urinary function probably via ellagic acid, a rich polyphenolic component of BRE. This mechanism of action by ellagic acid lowers blood dihydrotestosterone (DHT) levels by inhibiting the activity of the 5AR enzyme that produces DHT from testosterone [8]. Further, previous reports suggested that inhibitory properties of BRE improved prostate hypertrophy where ellagic acid act as an inhibitor of 5AR [22,31]. Therefore, BRE is believed to have contributed to the improvement in voiding symptoms observed in this study. 

The correlation between metabolic disease and prostate health has not been established conclusively until now. High cholesterol, a primary risk factor for cardiovascular disease, is also a risk factor for BPH, and cholesterol-lowering drugs are expected to help reduce BPH [5]. Recently, lower cholesterol levels have been suggested to reduce metabolic syndrome and hyperlipidemia, potentially improving prostate symptoms [39,40]. Here, supplementation of BRE significantly reduced serum TC and LDL-C levels, indicating improved prostate symptoms. In support, six-week animal studies with BRE improved blood cholesterol levels and BPH. Further, BRE supplementation lowers cholesterol levels by activating HMG-CoA reductase [8,9]. Additionally, other studies have shown that blackberry consumption improves lipid profiles [41,42]. These key observations suggest positive effects of BRE on lipid metabolism and prostate symptoms, and most observations of the study are consistent with previous clinical prognostic studies. Nevertheless, the mechanisms underlying these improvements are of considerable significance. Previous investigations with hormone-dependent prostate cancer cell lines (LNCaP) showed that BRE supplementation significantly decreased the expression of androgen-related genes, including androgen receptors (AR), prostate-specific antigens (PSA), and 5-alpha reductase 2. This reduction in gene expression leads to a decrease in prostate weight, epithelial cell thickness, and area of prostate follicles [8]. Moreover, ellagic acid may help reduce levels of DHT, a hormone that contributes to prostate enlargement [8]. Specifically, DHT strongly induces AR transcriptional activity [43], which profoundly affects gene regulation in prostate cells [44]. Ellagic acid can also inhibit cell proliferation and induce apoptosis in prostate cancer cells by regulating the Akt and mTOR pathways [45]. The findings suggest that ellagic acid is an important component of BRE that positively affects BPH and MCS. Furthermore, as a 5AR inhibitor, ellagic acid is the critical component in the underlying mechanism behind the BRE beneficial effects through various pathways, including Akt and mTOR pathways. Thus, BRE supplementation may alleviate BPH and voiding dysfunction in middle-aged and older men. However, additional research is necessary to fully understand the effects and potential benefits of ellagic acid supplementation on prostate health. This study suggests that improvements in voiding function are consistent with an inhibitory effect on prostate enlargement. Nonetheless, this study has its limitations. The small number of participants makes it difficult to generalize the results to other populations with BPH. Additionally, measures of urinary function did not include the urinary flow rate or prostate size. Therefore, it is necessary to validate the effectiveness of BRE in a large-scale human clinical trial involving individuals with prostate disorders.

## 5. Conclusions

A 12-week regimen of BRE supplementation in middle-aged and older men with climacteric syndrome was not effective in alleviating symptoms of andropause. However, it did result in reductions in TC and LDL-C levels in the blood, with no reported adverse effects. Moreover, improved IPSS scores suggested easier urination, indicating improved prostate health. As a result, we observed a significant improvement in the quality of life, as evidenced by the scores on the IPSS-QoL index. Overall, this study provides robust evidence supporting the safety of BRE as a functional food. Its supplementation potentially enhances lipid metabolism and alleviates dysuria symptoms, thus limiting the development of BPH.

## Figures and Tables

**Table 1:** Composition of the test product (BRE) and placebo products.

Ingredients	Test Tablet (%)	Placebo Supplement (%)
Unripe black berry extract (BRE)	62.5	-
Lactose mixed powder	10.6	31.65
Microcrystalline cellulose	5.38	23.25
Maltodextrin	10.63	35.42
HPMC	6.80	5.30
Silicon dioxide	1.00	1.00
CMC-CA	1.00	1.00
Magnesium stearate	0.70	0.70
Beet red pigment	0.20	0.20
Anhydrous citric acid	0.005	0.005
Titanium dioxide	0.60	0.60
Propylene glycol	0.59	0.57
Caramel coloring	-	0.30
Total	100	100

Abbreviations: HPMC, hydroxypropyl methylcellulose; CMC-CA, carboxymethylcellulose calcium.

**Table 2:** Demographic characteristics of the subjects.

	BRE Group (n = 15)	Placebo Group (n = 15)	Total (n = 30)	*p*-Value ^(1)^
Age (years)	66.13 ± 6.16	61.47 ± 7.65	63.80 ± 7.23	0.109
Height (cm)	167.27 ± 6.67	170.67 ± 6.84	168.97 ± 6.6	0.140
Weight (kg)	69.96 ± 9.26	75.35 ± 9.10	72.65 ± 9.43	0.159
BMI (kg/m^2^)	24.90 ± 1.95	25.79 ± 1.97	25.35 ± 1.98	0.350
SBP (mmHg)	124.27 ± 10.87	124.60 ± 13.80	124.43 ± 12.21	0.868
DBP (mmHg)	75.80 ± 11.26	76.60 ± 8.08	76.20 ± 9.64	0.999
Pulse (bpm)	67.33 ± 8.62	68.67 ± 10.95	68.00 ± 9.71	0.901
Total testosterone (ng/mL)	3.55 ± 0.73	3.40 ± 0.68	3.47 ± 0.69	0.383
SHBG (ng/mL)	47.2 ± 29.5	39.0 ± 11.5	43.1 ± 20.5	0.407
FT (ng/mL)	0.06 ± 0.01	0.06 ± 0.01	0.06 ± 0.01	0.619
BT (ng/mL)	1.43 ± 0.36	1.51 ± 0.38	1.47 ± 0.37	0.619
FSH (ng/mL)	14.1 ± 13.3	13.8 ± 14.1	14.0 ± 13.7	0.836
LH (ng/mL)	6.69 ± 5.19	6.46 ± 5.89	6.56 ± 5.54	0.351
HbA1c (%)	5.87 ± 0.31	5.71 ± 0.35	5.79 ± 0.34	0.268
Prolactin (ng/mL)	10.35 ± 4.81	9.83 ± 2.61	10.09 ± 3.81	0.934
Alcohol (Y/N)	8(53.3)/7(46.7)	5(33.3)/10(66.7)	13(43.3)/17(56.7)	0.423 ^(2)^
Drinking (years)	38.13 ± 11.6	36.0 ± 5.5	37.3 ± 9.5	0.495
Alcohol (unit ^(3)^/week)	5.03 ± 5.26	2.42 ± 2.15	4.02 ± 4.41	0.262
Smoking (Y/N)	1(3.3)/14(97.3)	0(0)/5(100)	1(3.3)/29(97.3)	0.999 ^(2)^
Smoking (pieces/day)	0.00 ± 0.00	12.5 ± 3.5	12.5 ± 3.5	-

Values are presented as mean ± SD or number (%). ^(1)^ Analyzed by Wilcoxon rank sum test compared between groups. ^(2)^ Analyzed by Fisher’s exact test. ^(3)^ Alcohol 1 unit = alcohol 10 g = alcohol 12.5 mL. Abbreviations: BRE, black berry extract; BMI, body mass index; SBP, systolic blood pressure; DBP, diastolic blood pressure; TT, total testosterone; FT, free testosterone; BT, bioavailable testosterone; SHBG, sex hormone binding globulin; FSH, follicle-stimulating hormone; LH, luteinizing hormone.

**Table 3:** Dietary intake of major nutrients and metabolic equivalent values of the subjects.

		BRE Group (n = 15)	Placebo Group (n = 15)	*p*-Value ^(2)^	*p*-Value ^(4)^
Energy (kcal)	Baseline	1486.15 ± 356.04	1742.39 ± 349.10	0.056	0.565
	6 week	1590.35 ± 450.47	1890.97 ± 410.07	0.678 (0.828)	
	Change from baseline	104.20 ± 345.20	148.58 ± 309.79		
	*p*-value ^(1)^	0.326	0.107		
	12 week	1496.95 ± 397.68	1908.68 ± 534.69	0.619 (0.374)	
	Change from baseline	10.81 ± 274.09	166.29 ± 587.85		
	*p*-value ^(1)^	>0.999	0.525		
	*p*-value ^(3)^	0.375	0.361	-	
CHO (g)	Baseline	237.35 ± 75.03	276.35 ± 53.25	0.042	0.624
	6 week	258.98 ± 71.00	295.85 ± 55.50	0.868 (0.380)	
	Change from baseline	21.62 ± 74.28	19.50 ± 60.41		
	*p*-value ^(1)^	0.296	0.252		
	12 week	250.64 ± 78.20	268.67 ± 43.12	0.648 (0.827)	
	Change from baseline	13.28 ± 53.74	−7.68 ± 61.86		
	*p*-value ^(1)^	0.626	0.934		
	*p*-value ^(3)^	0.464	0.236	-	
Lipids (g)	Baseline	32.72 ± 17.23	38.82 ± 21.64	0.281	0.327
	6 week	35.37 ± 23.36	45.52 ± 19.02	0.384 (0.689)	
	Change from baseline	2.66 ± 27.23	6.70 ± 26.09		
	*p*-value ^(1)^	0.808	0.188		
	12 week	31.05 ± 17.49	55.66 ± 42.94	0.534 (0.353)	
	Change from baseline	−1.67 ± 26.03	16.84 ± 51.40		
	*p*-value ^(1)^	0.855	0.359		
	*p*-value ^(3)^	0.805	0.324	-	
Protein (g)	Baseline	60.15 ± 15.79	70.62 ± 30.55	0.678	0.352
	6 week	65.47 ± 22.87	70.25 ± 20.76	0.740 (0.261)	
	Change from baseline	5.32 ± 24.52	−0.38 ± 36.04		
	*p*-value ^(1)^	0.807	>0.999		
	12 week	56.97 ± 15.48	80.41 ± 41.59	0.619 (0.670)	
	Change from baseline	−3.18 ± 17.19	9.79 ± 53.83		
	*p*-value ^(1)^	0.626	0.804		
	*p*-value ^(3)^	0.308	0.631	-	
Fiber (g)	Baseline	19.67 ± 6.86	20.05 ± 7.50	0.934	0.813
	6 week	22.40 ± 7.96	23.61 ± 8.12	0.836 (0.639)	
	Change from baseline	2.73 ± 5.85	3.56 ± 6.10		
	*p*-value ^(1)^	0.119	0.041		
	12 week	20.88 ± 7.40	22.87 ± 5.94	0.431 (0.620)	
	Change from baseline	1.21 ± 5.18	2.83 ± 8.49		
	*p*-value ^(1)^	0.391	0.252		
	*p*-value ^(3)^	0.202	0.195	-	
MET (min/week)	Baseline	2053.33 ± 1845.68	2120.00 ± 1998.40	0.405	0.403
	6 week	2277.33 ± 2460.27	3514.67 ± 3575.37	0.520	
	Change from baseline	224.00 ± 2709.47	1394.67 ± 2885.11		
	*p*-value ^(1)^	0.749	0.140		
	12 week	1826.67 ± 1766.59	2925.33 ± 2783.83	0.146	
	Change from baseline	−226.67 ± 1602.33	805.33 ± 2956.48		
	*p*-value ^(1)^	0.466	0.124		
	*p*-value ^(3)^	0.746	0.183	-	

Values are presented as mean ± SD. ^(1)^ Analyzed by Wilcoxon signed rank test compared within groups. ^(2)^ Analyzed by Wilcoxon rank sum test compared between groups (ANCOVA adjusted for baseline of carbohydrate. ^(3)^ Analyzed by RM-ANOVA compared within groups. ^(4)^ Analyzed by RM-ANOVA compared between groups. Abbreviations: CHO, carbohydrates; MET, metabolic equivalent task.

**Table 4:** Assessment of AMS, IPSS, and serum hormone levels in predetermined time during the 12-week intervention period.

	BRE Group (n = 15)				Placebo Group (n = 15)				
	Baseline	Week 6	Week 12	*p*-Value ^(1)^	Baseline	Week 6	Week 12	*p*-Value ^(1)^	*p*-Value ^(2)^
AMS total scores	45.3 ± 10.7	42.0 ± 8.1	38.3 ± 8.7	0.037	48.1 ± 8.0	41.0 ± 9.8 *	40.7 ± 8.1	0.001	0.967
Total IPSS	11.4 ± 3.3	9.0 ± 2.5 **	7.9 ± 6.4	0.047	10.8 ± 5.6	11.3 ± 6.9	9.7 ± 6.2	0.256	0.064
Storage symptomssub-score	4.0 ± 2.4	3.5 ± 1.7	3.2 ± 2.9	0.312	4.5 ± 1.5	4.7 ± 2.2	4.2 ± 1.9	0.406	0.526
Voiding symptoms sub-score	7.4 ± 2.0	5.5 ± 2.2 **	4.7 ± 4.5	0.023	6.3 ± 4.7	6.6 ± 5.4	5.5 ± 5.0	0.010	0.039
IPSS-QoL	3.1 ± 0.8	2.9 ± 1.1	2.2 ± 1.2	0.047	3.2 ± 1.4	2.9 ± 1.4	2.9 ± 1.2	0.281	0.275
Total testosterone (ng/mL)	3.55 ± 0.73		3.44 ± 0.79	0.538	3.40 ± 0.68		3.37 ± 0.78	0.772	0.999
SHBG (ng/mL)	47.17 ± 23.26		44.27 ± 23.26	0.241	38.96 ± 11.54		37.71 ± 13.19	0.679	0.481
FT (ng/mL)	0.06 ± 0.01		0.06 ± 0.02	0.903	0.06 ± 0.01		0.06 ± 0.01	0.934	0.836
BT (ng/mL)	1.43 ± 0.36		1.47 ± 0.44	0.999	1.51 ± 0.38		1.52 ± 0.35	0.993	0.999
FSH (ng/mL)	14.0 ± 13.29		12.73 ± 11.14	0.173	13.78 ± 14.14		13.13 ± 14.23	0.086	0.846
LH (ng/mL)	6.69 ± 4.82		6.65 ± 5.19	0.987	6.83 ± 6.64		6.46 ± 5.89	0.710	0.648

Values are presented as means ± SD. ^(1)^ Analyzed by Wilcoxon signed rank test compared within groups. ^(2)^ Analyzed by Wilcoxon rank sum test compared between groups. *: *p* < 0.05, **: *p* < 0.01 (baseline vs. week 6) by Wilcoxon signed rank test compared within groups. Abbreviations: AMS, aging males’ symptoms; IPSS, international prostate symptom score; IPSS-QoL, IPSS quality of life; TT, total testosterone; FT, free testosterone; BT, bioavailable testosterone; SHBG, sex hormone binding globulin; FSH, follicle-stimulating hormone; LH, luteinizing hormone.

**Table 5:** Anthropometric indices and lipid profiles obtained before and after treatment.

	BRE Group (n = 15)				Placebo Group (n = 15)				
	Baseline	Week 12	Change	*p*-Value ^(1)^	Baseline	Week 12	Change	*p*-Value ^(1)^	*p*-Value ^(2)^
Weight (kg)	70.0 ± 9.3	70.0 ± 8.7	0.0 ± 1.4	0.879	75.4 ± 9.1	75.8 ± 9.5	0.5 ± 1.3	0.210	0.917
BMI (kg/m^2^)	24.9 ± 2.0	25.0 ± 2.0	0.1 ± 0.6	0.692	25.8 ± 2.0	25.9 ± 2.1	0.2 ± 0.5	0.253	0.999
BFM (kg)	18.0 ± 5.0	18.3 ± 4.9	0.3 ± 1.2	0.435	17.9 ± 4.2	18.3 ± 4.4	0.4 ± 1.3	0.234	0.561
PBF (%)	25.5 ± 5.4	25.9 ± 5.3	0.4 ± 1.3	0.221	23.6 ± 3.7	24.0 ± 0.9	0.4 ± 1.4	0.235	0.724
FFM (kg)	51.9 ± 6.2	51.6 ± 5.7	−0.3 ± 1.0	0.332	57.4 ± 6.1	575 ± 6.4	0.03 ± 1.0	0.923	0.395
WC (cm)	89.5 ± 7.3	90.1 ± 7.1	0.6 ± 1.7	0.170	91.7 ± 6.0	91.6 ± 6.0	−0.01 ± 1.6	0.911	0.271
HC (cm)	94.1 ± 4.7	95.1 ± 5.1	0.9 ± 1.6	0.025	96.8 ± 3.8	97.5 ± 3.7	0.7 ± 0.7	0.003	0.587
WHR	0.97 ± 0.05	0.95 ± 0.04	−0.02 ± 0.04	0.287	0.95 ± 0.04	0.94 ± 0.04	−0.01 ± 0.02	0.109	0.445
TC (mg/dL)	199.3 ± 42.3	185.7 ± 39.5	−13.6 ± 30.4	0.079	177.6 ± 37.6	186.5 ± 36.8	8.9 ± 15.7	0.044	0.011
TG (mg/dL)	149.0 ± 59.6	179.7 ± 83.1	30.7 ± 77.2	0.492	162.3 ± 75.5	143.1 ± 39.5	−19.2 ± 66.5	0.463	0.319
HDL-C (mg/dL)	49.2 ± 11.7	49.6 ± 13.6	0.40 ± 5.7	0.528	41.3 ± 7.7	44.9 ± 8.5	3.6 ± 5.9	0.012	0.307
LDL-C (mg/dL)	120.3 ± 38.0	100.1 ± 29.8	−20.1 ± 34.7	0.049	105.7 ± 30.5	113.0 ± 31.4	7.3 ± 13.3	0.083	0.007
LDL-C/HDL-C	2.55 ± 0.81	2.13 ± 0.76	−0.41 ± 0.62	0.017	2.56 ± 0.62	2.52 ± 0.67	−0.04 ± 0.40	0.934	0.062
TC/HDL-C	4.19 ± 0.34	3.94 ± 1.10	−0.25 ± 0.81	0.217	4.32 ± 0.70	4.19 ± 0.74	−0.13 ± 0.57	0.639	0.589

Values are presented as means ± SD. ^(1)^ Analyzed by Wilcoxon signed rank test compared within groups. ^(2)^ Analyzed by Wilcoxon rank sum test compared between groups (ANCOVA adjusted for baseline of HDL-C). Abbreviation: BMI, body mass index; BFM, body fat mass; PBF, percent body fat; FFM, fat free mass; WC, waist measurement; HC, hip measurement; WHR, waist–hip ratio, TC, total cholesterol; TG, triglycerides; LDL-C, low-density lipoprotein-cholesterol; HDL-C, high-density lipoprotein-cholesterol.

## Data Availability

The datasets generated during and/or analyzed during the current study are available from the corresponding author on reasonable request.

## Supplementary Materials

The following supporting information can be downloaded at: https://www.mdpi.com/article/10.3390/nu15153313/s1, Figure S1: Schematic diagram showing volunteer recruitment process in the trial; Table S1: Laboratory profiles of the subjects in the study.
