# Supplementary material for: Effects of Unripe Black Raspberry Extract Supplementation on Male Climacteric Syndrome and Voiding Dysfunction: A Pilot, Randomized, Double-Blind, Placebo-Controlled Trial
Source: Nutrients. 2023 Jul 26;15(15):3313. doi: 10.3390/nu15153313 (PMC10421075; doi:10.3390/nu15153313)
Supplement: Supplementary file 1 [file nutrients-15-03313-s001.zip › nutrients-2484939-supplementary.pdf]

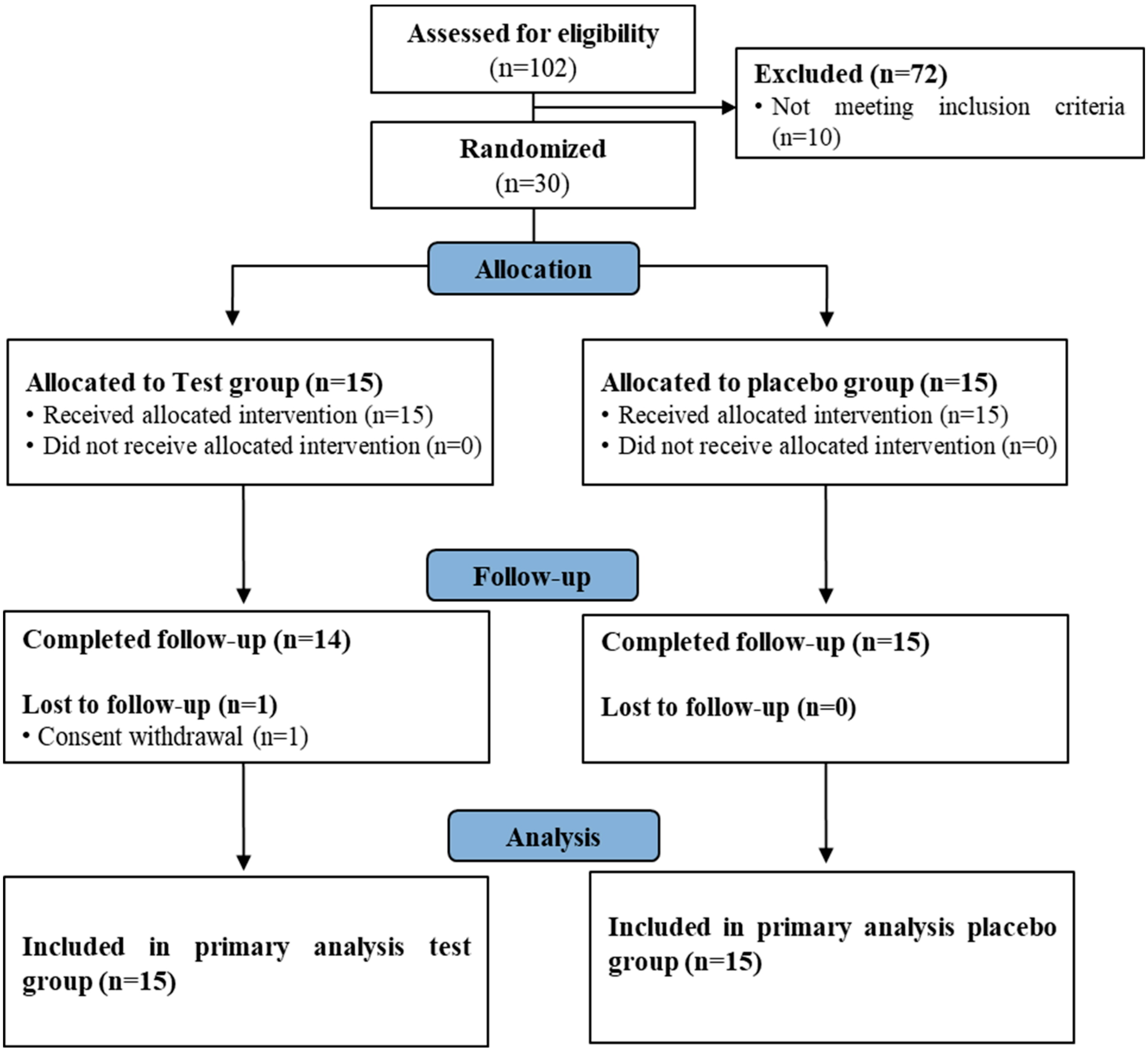

Supplementary Figure S1. Schematic diagram showing volunteer recruitment process in the trial

Supplementary Table S1. Laboratory profiles of the subjects in the study

Laboratory profiles (Standard range)	BRE group (n=15)			Placebo group (n=15)			
	Baseline	Week 12	p-value [1)]	Baseline	Week 12	p-value [1)]	p value[2)]
WBC (4.8–10.8×10³/μL)	5.5±1.3	5.4±1.2	0.903	5.7±1.2	5.4±0.7	0.268	0.407
RBC (4.2–5.4×100³/μL)	4.6±0.4	4.6±0.4	0.959	4.56±0.4	4.6±0.4	0.119	0.290
Hemoglobin (12–16g/dL)	14.5±0.9	14.5±1.0	0.699	14.1±0.9	14.3±0.9	0.072	0.663
Hematocrit (37–47%)	42.8±2.6	43.2±2.5	0.508	42.4±2.7	42.7±2.3	0.173	0.575
Platelet (130–450×10³/μL)	232.1±31.3	246.3±46.4	0.028	206.1±39.3	215.1±30.2	0.117	0.885
ALP (45~129 IU/L	67.8±22.8	71.7±27.8	0.065	68.3±14.6	67.8±15.5	0.971	0.220
T_PSA (≤4 ng/ml)	0.94±0.50	1.38±1.44	0.051	1.32±0.76	1.49±1.23	0.793	0.340
Sg (1.005~1.030)	1.0±0.0	1.0±0.0	0.325	1.0±0.0	1.0±0.0	0.932	0.442
hs-CRP (~5mg/L)	0.7±0.9	0.7±0.9	0..999	0.5±0.6	0.9±1.4	0.229	0.361
GGT (8–48IU/L)	33.4±17.1	31.0±16.9	0.210	22.5±8.4	19.5±11.2	0.130	0.868
AST (12–33IU/L)	28.2±4.3	26.1±3.3	0.138	26.7±8.2	24.9±6.8	0.596	0.693
ALT (5–35IU/L)	31.6±19.2	26.3±11.4	0.026	30.7±19.5	25.9±7.6	0.281	0.454
Total bilirubin (0.2–1.2mg/dL)	0.9±0.3	0.9±0.3	0.417	1.0±0.3	0.9±0.2	0.609	0.384
Total protein (6.7–8.3g/dL)	7.1±0.3	7.1±0.3	0.363	7.2±0.2	7.1±0.3	0.802	0.094
Albumin (3.5–5.3g/dL)	4.5±0.2	4.6±0.1	0.256	4.6±0.2	4.6±0.2	0.988	0.453
BUN (8–23mg/dL)	17.7±4.2	17.2±5.6	0.750	18.1±3.4	17.3±400	0.365	0.631
Creatinine (0.7–1.7mg/dL)	0.9±0.1	0.9±0.2	0.750	0.9±0.2	0.9±0.2	0.732	0.693
Glucose (74–106mg/dL)	91.7±12.0	94.0±11.8	0.146	91.3±6.8	90.1±8.6	0.418	0.145
CK (50~200 IU/L)	120.7±58.9	129.2±64.7	0.727	122.3±66.8	131.0±69.6	0.325	0.604
LDH (218~472 IU/L)	372.1±43.2	366.5±35.3	0.749	380.3±43.7	355.7±45.9	0.027	0.135
pH(urine) (4.5~9.0)	6.3±1.2	6.4±1.1	0.871	6.2±1.1	6.3±0.8	0.732	0.689

Values are presented as mean ± SD. [1)] Analyzed by paired *t* test. [2)] Analyzed by Linear Mixed Model between groups
Abbreviations: Abbreviation: WBC, white blood cell; RBC, red blood cell; Hb, hemoglobin; Hct, hematocrit; PLT, platelet; ALP, alkaline phosphatase; GGT, gamma-glutamyltransferase; AST, aspartate transaminase; ALT, alanine transaminase; BUN, blood urea nitrogen; CK, creatine kinase; LD, lactate dehydrogenase; hs-CRP, high-sensitive C-reactive protein.
